# The Mountain Meadows Massacre and “poisoned springs”: scientific testing of the more recent, anthrax theory

**DOI:** 10.1007/s00414-012-0681-y

**Published:** 2012-03-07

**Authors:** Ugo A. Perego, Alessandro Achilli, Jayne E. Ekins, Lucio Milani, Martina Lari, Elena Pilli, Alexis Brown, Erin P. Price, Spenser R. Wolken, Molly Matthews, Christina A. Allen, Talima R. Pearson, Norman Angerhofer, David Caramelli, Tim Kupferschmid, Paul S. Keim, Scott R. Woodward

**Affiliations:** 1 Sorenson Molecular Genealogy Foundation, 2480 South Main Street #200, Salt Lake City, UT 84115 USA; 2 Dipartimento di Genetica e Microbiologia, Università di Pavia, Via Ferrata 1, 27100 Pavia, Italy; 3 Dipartimento di Biologia Cellulare e Ambientale, Università di Perugia, Via Elce di Sotto, 06123 Perugia, Italy; 4 Dipartimento di Biologia Evoluzionistica, Università di Firenze, Via del Proconsolo 12, 50122 Firenze, Italy; 5 Sorenson Forensics, 2495 South West Temple, Salt Lake City, UT 84115 USA; 6 Center for Microbial Genetics and Genomics, Northern Arizona University, P.O. Box 4073, Flagstaff, AZ 86011 USA; 7 Translational Genomics Research Institute, 445 North Fifth Street, Phoenix, AZ 85004 USA

**Keywords:** Anthrax, *Bacillus anthracis*, Mitochondrial DNA, Ancient DNA

## Abstract

It has been recorded that one of the possible causes that eventually escalated into the 1857 manslaughter at Mountain Meadows in Southern Utah was the poisoning of an open spring by the Fancher–Baker party as they crossed the Utah territory on their way from Arkansas to California. Historical accounts report that a number of cattle died, followed by human casualties from those that came in contact with the dead animals. Even after the Arkansas party departed, animals continued to perish and people were still afflicted by some unknown plague. Proctor Hancock Robison, a local 14-year-old boy, died shortly after skinning one of the “poisoned” cows. A careful review of the historical records, along with the more recent scientific literature, seems to exclude the likelihood of actual poisoning in favor of a more recent theory that would point to the bacterium *Bacillus anthracis* as the possible cause of human and animal deaths. In order to test this hypothesis, Proctor’s remains were exhumed, identified through mitochondrial DNA analysis, and tested for the presence of anthrax spores. Although preliminary testing of remains and soil was negative, description of the clinical conditions that affected Proctor and other individuals does not completely rule out the hypothesis of death by anthrax.

**Electronic supplementary material:**

The online version of this article (doi:10.1007/s00414-012-0681-y) contains supplementary material, which is available to authorized users.

## Introduction

On 11 September 1857, approximately 120 men, women, and children fell victim to one of the largest domestic acts of violence ever witnessed on Northern American soil [1]. Local residents of southern Utah aided by Southern Paiute Indians carried out the mass slaughter of the Fancher–Baker wagon train: a group of emigrants on their way from Arkansas to California. Only 17 children age six and younger were spared [2]. On 29 September 1857, when Utah Territorial militiaman John D. Lee reported the Mountain Meadows Massacre to civil and church authorities governing the Utah territory from Salt Lake City, he demonized the Arkansas emigrants, telling that they had deliberately poisoned cattle and an open spring near Fillmore, Utah [3]. The poisoning, Lee said, led to the death of local Indians and civilians. One Fillmore resident, a 14-year-old boy named Proctor Hancock Robison, was confirmed to have died on 21 September 1857 shortly after skinning the carcass of a “poisoned” cow [4]. For more than a century, the unlikely poisoning theory continued in some lines of Utah historiography as a key event that instigated the barbaric killings that followed. However, a more recent interpretation argues that the cattle and humans were not the victims of poisoning, but of anthrax, a lethal disease unknown in 1857, caused by the spore-forming bacterium *Bacillus anthracis* [5].

If not promptly treated with antibiotics such as penicillin, anthrax can result in the death of 10–40% of patients and is fatal within as few as 24 h [6]. The most common of the three forms of this disease is cutaneous—with respiratory and gastrointestinal being the other two—having as clinical manifestations increased body temperature, and bullous lesions resulting in severe erythema, edema, and tissue necrosis [6, 7]. The disease is transmitted by contact or inhalation of the anthrax spores from animal to animal, or from infected animals to humans, but it is seldom contagious between humans [6]. Normally, such lesions occur on environmentally exposed areas of the body such as the face, neck, chest, and the upper limbs [7].

The description of anthrax symptoms and incubation time fit with the historical accounts describing the events that lead to the sickness and death of both livestock and humans, including Proctor Hancock Robison. In fact, it is reported that while the 14-year-old was skinning one of the dead cattle, he scratched a sore on his nose, and within hours, his face began to swell greatly until his eyes were fully shut [4]. The cutaneous edema that affected the teenage boy was so extensive that he was unrecognizable. Proctor’s misery came to an end shortly after contracting the deadly pathogen. Although intoxication by severe poisoning could have also resulted in rapid death, it is highly unlikely that this was what caused Proctor’s fate. A considerable amount of any toxic substance is necessary to poison an open source of water and thus cause serious health consequences in both animals and humans. Furthermore, poisons are mostly fatal when ingested and not necessarily through skin absorption as would have been the case with Proctor and others who only touched the carcasses. In addition, poisons such as arsenic—the toxic substance mentioned in the historical records [4]—would affect the gastrointestinal system, resulting in severe stomach cramping, vomiting, and loss of liquids through uncontrolled bowel movements [8]. The case of the young man from Fillmore presented clinical conditions that strongly suggest cutaneous anthrax as the likely cause of his death. This disease was unknown, yet endemic in North America during the nineteenth century, and its etiology was often mistaken for something else. To corroborate on the death-by-anthrax theory, an account has been located mentioning the death of an unnamed member of the Fancher–Baker caravan shortly before the group reached the Utah territory. The newspaper entry reported that “one of the leaders from Arkansas was bitten in the hand while asleep by a tarantula” and died in much suffering shortly thereafter [9]. Bullous lesions with necrosis of the surrounding tissues caused by cutaneous anthrax could have been easily mistaken as a spider bite [6], particularly if such pathology was unknown. Few spider species worldwide are known to cause medically significant consequences from biting, and tarantulas are not one of them [10]. By request of the Robison family, Proctor’s burial site was located and his remains exhumed and identified to investigate the cause of his death.

## Materials and methods

### Exhumation

Legal permits and location of the gravesite within the old Fillmore cemetery for the exhumation of Proctor Hancock Robison was obtained by his family. The gravesite is currently marked by a modern tombstone. There were uncertainties pertaining to the actual location of Proctor’s resting place, so to confirm the identity of the remains, we employed the assistance of Sorenson Forensics for the exhumation process while the ancient DNA testing was carried out by the Evolutionary Biology Department at the University of Florence in Italy.

According to cemetery records, the gravesite is located on the east side of the tombstone, on the opposite side of where the engravings are located. An area of 1.20 m wide by 2.50 m long was selected for the excavation. Approximately 20 cm under the surface, what appeared to be the original grave marker was recovered with the epitaph “Proctor Hancock Robison” carved in large capital letters. At about 1.45 m in depth, we began recovering small pieces of wood of what was left of the original coffin. Bones were found at a depth of 1.60 m, with the skeleton discovered in fetal position, however, the skull and jaw were found at approximately half a meter west of the body.

A 5 × 2.5-cm section of each femur was carefully removed for DNA extraction and mitochondrial DNA (mtDNA) testing for identification confirmation purposes. One small rib and two molars were also collected as backup specimens in case of poor DNA quality from the first sample. Buccal swabs were previously collected from three maternal relatives of Proctor Hancock Robison (Fig. S1). DNA from these modern samples were extracted and sequenced at Sorenson Genomics Laboratories, where control region data for hypervariable segment (HVS)-I and HVS-II (from np 16001 to np 16569 and from np 1 to np 590) was produced. MtDNA haplotypes for the three individuals handling the bones were also available to exclude contamination issues. A total of 65 soil samples were collected in proximity of the skull, upper torso, and pelvic region, as these areas are likely candidates for the presence of any *B. anthracis* spores, residual from likely epidermis or bowel discharges at the time of Proctor’s passing and burial.

### DNA extraction

DNA was extracted from two of the bone fragments obtained, respectively, from the rib and a section of the femur. To prevent contamination from prior handling, the outer layer of each bone was removed with a rotary tool and was then irradiated (1 h under UV light for each side) and powdered.

The powder was incubated for 72 h with 0.5 M EDTA then digested overnight with proteinase K and SDS; afterwards, DNA was extracted by means of a phenol–chloroform protocol and subsequent silica-based purification [11, modified]. Multiple negative controls were included in each extraction [12–14].

### Amplification of mtDNA

We amplified mtDNA as previously described [15]. An additional primer pair, L029/H180, was used to amplify a 197-bp long fragment of HVS-II. Each extract was amplified at least twice for each primer pair.

### Cloning and sequencing

PCR products were cloned and sequenced according to previously described methods [16].

### *B. anthracis* testing

Sixty-five soil samples and four non-soil samples were collected at graveside and sent to the Center for Microbial Genetics and Genomics in Arizona for *B. anthracis* detection (Table S1). Eleven soil samples (1, 5, 10, 19, 23, 30, 34, 40, 46, 57, and 64) were randomly chosen for DNA extraction using a soil DNA extraction kit (MO-BIO, Carlsbad, CA). Approximately 5 g of soil was used for extractions. These DNA extractions were subjected to (a) a 16S ribosomal RNA (detects all bacteria) real-time PCR assay to confirm extraction efficiency of bacterial cells and (b) the *plcR* SYBR real-time PCR assay [17] to detect the presence of *B. anthracis* DNA.

**Fig. 1:**
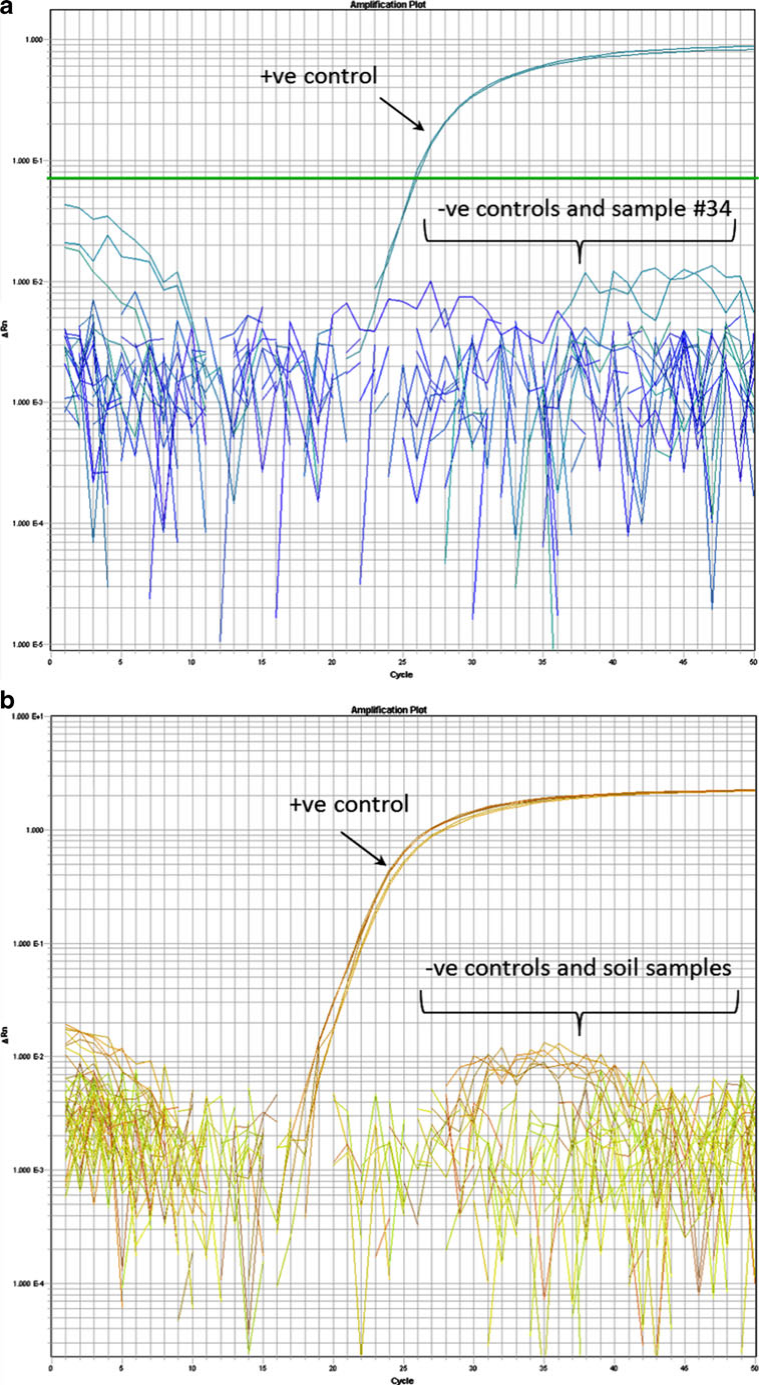
Real-time PCR images of the PHR gravesite soil sample #34 (**a**) and #19, #23, #57, and #64 (**b**). The real-time PCR targeted the *B. anthracis*-specific target, *plcR* [17]. Note that there was no amplification detected for any of these samples

In the first heat treatment experiment, approximately 1–3 g soil from samples 19, 30, and 34 were placed into 1 mL Luria Bertani (LB) broth (Becton-Dickinson, Franklin Lakes, NJ) in Eppendorf tubes, vortexed vigorously, and subjected to increasing heat exposure. We used heat treatment in order to destroy non-sporulating bacteria, thereby selecting for sporulated *B. anthracis* and reducing background contaminant growth. The 15 tubes were placed into a hot water bath at 91°C. A single tube for each sample was taken out consecutively every 5 min over a period of 25 min. The samples consisted of 5, 10, 15, 20, and 25 min heat sterilization periods. Fifty microliters of each sample was pipetted onto 5% sheep blood agar (SBA; Hardy Diagnostics, Santa Maria, CA) for each of the heat sterilization periods and incubated at 37°C for 18 h. Suspect *B. anthracis* colonies (Fig. S2) were DNA extracted using heat soak treatment and were genotyped using the *plcR* real-time PCR assay (Fig. 1).

We greatly reduced the heat treatment of the soils in our second experiment due to negative results in the first heat treatments. Six randomly chosen soil samples (5, 9, 57, 19, 23, and 64) were subjected to heat treatment at 70°C for 5 min. Approximately 1–3 g soil was treated as described in the first experiment. Following treatment, these samples were plated onto SBA and polymyxin B–lysozyme–EDTA–thallous acetate (PLET) agar (Hardy Diagnostics), the latter of which is more selective for the cultivation of *B. anthracis* than other agars [18]. SBA plates were incubated at 37°C for 18 h, whereas PLET agar plates were incubated for approximately 36 h. Suspect colonies (Fig. S2) were extracted using heat soak treatment and were genotyped using the *plcR* real-time PCR assay (Fig. 1).

The next experiment involved culturing of *B. anthracis* directly from soil samples using 70% ethanol (EtOH). EtOH has been used as an alternative to heat treatment for the selection of sporulating bacteria, as it destroys vegetative cells [19]. We EtOH-treated samples (5, 9, 57, 19, 23, and 64) by placing approx. 5 g soil into ∼15 mL 70% EtOH. The samples were vigorously vortexed and then allowed to evaporate overnight. The soil material was resuspended in Luria Bertani broth and again plated onto SBA and PLET. Suspect colonies were extracted using heat soak treatment and were genotyped using the *plcR* real-time PCR assay. In a final attempt to obtain *B. anthracis* from the gravesite samples, we decided to focus on bone due to the hypothesized higher probability of finding bacteria in this material [20].

The three bone samples (rib, facial bones, and “inside the cheek”) were manually broken up into smaller pieces in order to expose marrow. The coffin wood sample was also broken up manually. These samples were placed into 25 mL LB broth and allowed to incubate at 37°C for 48 h. In the first plating attempt, 1 mL material from each of the four samples was plated onto SBA, PLET agar, and LB agar (the latter of which is nonselective) and incubated for approximately 18 h. Due to the large number of colonies observed in this first attempt, we plated 25 μL of material onto PLET agar and incubated for approximately 20 h to obtain individual colonies. From the first plating attempt, approximately 10 μL confluent bacterial culture was subjected to heat soak treatment. From the second plating attempt, only colonies suspected of being *B. anthracis* were picked and extracted using heat soak treatment.

## Results and discussion

### Ancient DNA

Comparative analysis of mtDNA results for three living maternal relatives of Proctor Hancock Robison produced a positive identification of the exhumed remains. MtDNA control region sequences were obtained for three living relatives sharing a common maternal ancestry with Proctor Hancock Robison’s mother, Lucretia Proctor Hancock (Fig. S1). The three haplotypes were identical (Table 1) and matched three (16126C, 16294T, and 16304C) and two (73G and 195C) transitions observed in the sequence obtained from Proctor’s shorter HVS-I and HVS-II fragments, respectively. Mutations 16126C, 16294T, 16304C, and 73G are part of the basal Western Eurasian mtDNA haplogroup T2b motif [21]. A query of the mtDNA control region database at the Sorenson Molecular Genealogy Foundation (∼82,000 haplotypes; www.smgf.org) revealed that the complete haplotypes for the living relatives was found in only two individuals, also sharing Lucretia Proctor Hancock as a common maternal ancestor. The limited Proctor’s haplotype was shared with nine additional individuals. A similar query using the EMPOP database (www.empop.org) yielded no identical matches for both haplotypes. These queries suggest that the mtDNA profiles retrieved for the living individuals tested in the current study and from the Fillmore gravesite are indeed rare in the general population, corroborating the genealogical data available for this family. Such evidence strongly supports that the skeletal remains recovered at the Fillmore cemetery are indeed those of Proctor Hancock Robison. Additionally, the three scientists that worked on the excavation and on the DNA extraction belong to two separate and distinctive haplogroups, thus excluding the possibility of contamination by modern DNA.

**Table 1:** Mitochondrial DNA haplotypes for the four individuals tested in this study, including the ancient remains for Proctor Hancock Robison (PHR)

ID	Haplotype	Range sequenced
1	16126C, 16294T, 16304C, 16445C, 16519C, 73G, 195C, 263G, 309.1C, 315.1C, 321C	16001–590
2	16126C, 16294T, 16304C, 16445C, 16519C, 73G, 195C, 263G, 309.1C, 315.1C, 321C	16001–590
3	16126C, 16294T, 16304C, 16445C, 16519C, 73G, 195C, 263G, 309.1C, 315.1C, 321C	16001–590
PHR	16126C, 16294T, 16304C,……………………….73G, 195C,………………………………………	15995–16402; 7–203

### *B. anthracis* detection

We used several approaches for identifying *B. anthracis* spores in soil and bone material from the Fillmore gravesite. First, we attempted to detect *B. anthracis* DNA in soil samples by performing extractions directly on several soil specimens obtained from different areas of the gravesite. We verified that our soil DNA extraction was successful based on a positive 16S RNA PCR; however, all soil samples were negative for *plcR* (Fig. 1), indicating that no *Bacillus* species (including *B. anthracis*) DNA could be identified. Our subsequent attempts to detect *B. anthracis* spores in soil and bone samples employed heat and desiccation treatments, followed by selective agar plating. However, all attempts to detect viable *B. anthracis* spores were negative based on the *plcR* assay. This result was not entirely surprising as we found it difficult to select for and identify suspect colonies of *B. anthracis* using SBA and PLET agar due to the high amount of background growth.

## Conclusion

In 1857, following the illness and death of cattle, Paiute Indians, and local residents near Fillmore, Utah, militiaman John D. Lee began spreading the rumor that members of the Fancher–Baker train wagon, on their way from Arkansas to California, purposely poisoned a spring in retaliation toward the unfavorable dealings experienced with Utah territory citizens. Although the sickness and life toll was real, it is highly unlikely that poisoning was the cause of this epidemic. However, this important event had historical and sociopolitical implications as it is possible that it was used as one of the excuses for the execution of nearly all members of the Fancher–Baker group on 11 September 1857 in the area of Mountain Meadows. A careful review of the historical accounts that have survived to our day seems to indicate that anthrax and not poisoning was the likely source of the fatalities near Fillmore. DNA testing was carried out successfully to identify Proctor Hancock Robison, a 14-old boy who died on 21 September 1857 after coming in contact with one of the contaminated carcasses. Proctor’s remains and samples from the soil surrounding his bones have been tested for the presence of *B. anthracis* and to validate the hypothesis that anthrax, which was unknown at that time, was the cause of the boy’s illness.

Based on our consistently negative PCR and culture results, we were unable to demonstrate that *B. anthracis* was present in the gravesite soil, bone, or coffin wood samples. Given our focus on culturing *B. anthracis*, we feel reasonably confident that viable *B. anthracis* cells are not present in these samples or are present in too low a concentration to be detected using our techniques.

Our task of isolating viable *B. anthracis* spores from a case of anthrax that occurred approximately 150 years ago was non-trivial. First, the soil microbiota harbors the highest population of prokaryotes on earth [22]; thus, identifying 150-year-old spores in soil is complicated by the presence of an enormous and dynamic microbial load. Second, the age of potential *B. anthracis* spores in the grave samples may have rendered them considerably more susceptible to the heat and EtOH treatments than young cultures. Third, the gravesite environment may not have been conducive to long-term spore survival or may have, over time, concentrated spores in regions not sampled in the current study [23].

Despite our negative detection results, we cannot definitely rule out the absence of *B. anthracis* at or surrounding the Proctor gravesite. Previous studies have yielded positive *B. anthracis* spore resuscitation from ancient material, including 60-year-old soil samples [24] and, somewhat contentiously, 200-year-old bones from Kruger National Park [25, 26]. Similarly, DNA from the causative agent of plague, *Yersinia pestis*, has been isolated from ancient soft tissue specimens thought to be 400 years old [20]. Based on the success of detecting pathogen in these earlier studies, the Proctor gravesite samples may still yield positive *B. anthracis* detection. In the current study, we only used a small amount of soil (∼5 g) for our DNA extractions. Therefore, processing greater volumes of soil may yield positive results, including sampling soil from low-lying areas around the gravesite, which may improve probability of detection [23]. However, based on previous work on ancient pathogen detection and our consistently negative culturing results, the most fruitful approach would be to attempt *B. anthracis* DNA extractions on the recovered bone fragments.

## Electronic supplementary material

Below is the link to the electronic supplementary material.

ESM 1 (DOC 1,996 kb)

ESM 2 (TXT 7 kb)

ESM 3 (TXT 3 kb)
