# Supplementary material for: The Mountain Meadows Massacre and “poisoned springs”: scientific testing of the more recent, anthrax theory
Source: Int J Legal Med. 2012 Mar 7;127(1):77–83. doi: 10.1007/s00414-012-0681-y (PMC3538018; doi:10.1007/s00414-012-0681-y)

**Supplemental Online Materials**

**Table S1.** List of samples (location and type) collected at the Proctor H. Robison’s gravesite at the Fillmore, Utah cemetery.

| Original ID | NAU ID | Sample Description | Approx. Quantity |
| --- | --- | --- | --- |
| **Soils:** |  |  |  |
| 1 | PHR 1 | Soil 56in. down, 36in. from the south flag where first wood was found | 50mL conical |
| 2 | PHR 2 | Soil collected with soil sampler approx. 4in. below soil sample 1 | 50mL conical |
| 3-6 | PHR 3-6 | Soil around and on top of first bone found | 50mL conical |
| 7-9 | PHR 7-9 | Soil around edges of 1^st^ bone found prior to removal of bones from grave | 50mL conical |
| 10-13 | PHR 10-13 | Soil under and around pelvis | 50mL conical |
| 14 | PHR 14 | Null | 50mL conical |
| 15-17 | PHR 15-17 | Soil alongside femur | 50mL conical |
| 18-19 | PHR 18-19 | Soil around lower thoracic region of bones | 50mL conical |
| 20-23 | PHR 20-23 | Soil from inside pelvis | 50mL conical |
| 24 | PHR 24 | Null | 50mL conical |
| 25-26 | PHR 25-26 | Soil around sacrum | 50mL conical |
| 27-30 | PHR 27-30 | Soil around upper thoracic region of bones | 50mL conical |
| 31-33 | PHR 31-33 | Soil from inside of lower jaw | 50mL conical |
| 34-43 | PHR 34-43 | Soil around skull | 50mL conical |
| 44-45 | PHR 44-45 | Soil scraped off lower jaw | 50mL conical |
| 46-50 | PHR 46-50 | Soil around skull | 50mL conical |
| 51-65 | PHR 51-65 | Soil from area where skull was removed from grave | 50mL conical |
| **Non-soils:** |  |  |  |
| A | PHR A | Coffin wood | 50mL conical |
| E | PHR E | Rib #1 collected from grave of PHR at Filmore, UT City Cemetery, 21Oct08 | Approx. 6in. fragment |
| J | PHR J | Facial bones | 3-4 pieces @ 1in. long |
| I | PHR I | “Inside the Cheek” | 20mL of wood fragments |

**Figure S1.** Genealogical relationship between Proctor Hancock Robison and three individuals sharing the maternal line back to his mother Lucretia P. Hancock.


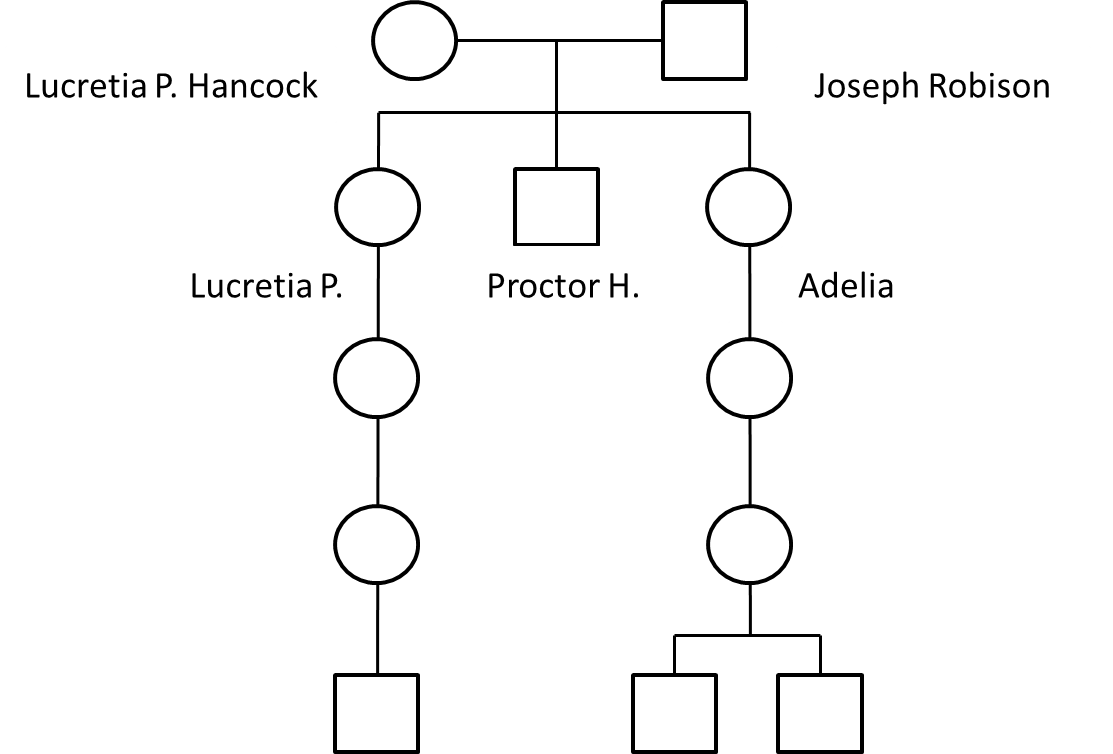


**Figure S2.** Example PLET (polymyxin B, lysozyme, EDTA, thallium acetate) agar growth for the PHR grave samples 18 and 46 (LHS), and 57 and 10 (RHS). Suspect *B. anthracis* colonies were picked and DNA-extracted for testing against the *plcR* assay. No colonies were *B. anthracis* according to this assay.


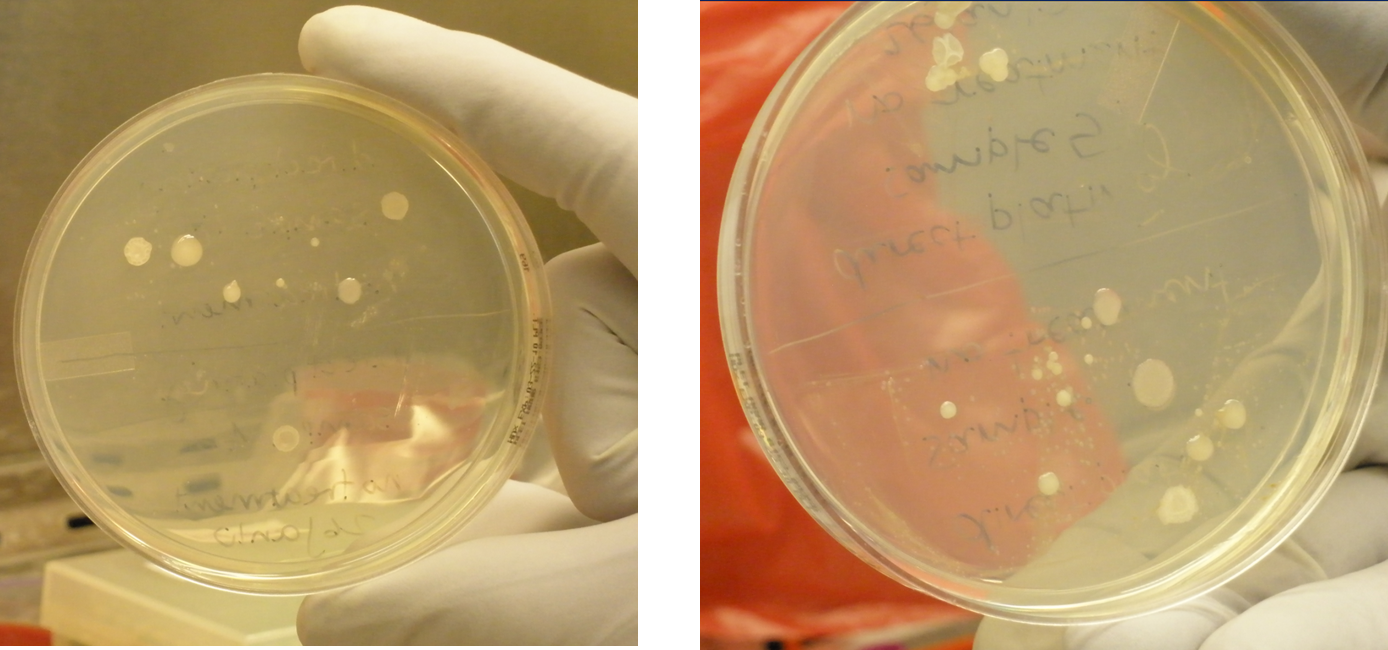

Supplement: Supplementary file 1 — (DOC 1,996 kb) [file 414_2012_681_MOESM1_ESM.doc]
